# Development of liver surface nodularity quantification program and its clinical application in nonalcoholic fatty liver disease

**DOI:** 10.1038/s41598-019-46442-y

**Published:** 2019-07-10

**Authors:** Tae-Hoon Kim, Ji Eon Kim, Jong-Hyun Ryu, Chang-Won Jeong

**Affiliations:** Medical Convergence Research Center, Wonkwang University, Iksan, 54538 Republic of Korea

**Keywords:** Experimental models of disease, Software

## Abstract

The liver morphological changes in relation to fibrosis stage in nonalcoholic fatty liver disease (NAFLD) have not yet been clearly understood. This study was to develop a liver surface nodularity (LSN) quantification program and to compare the fibrosis grades in simple steatosis (SS) and nonalcoholic steatohepatitis (NASH). Thirty subjects (7 normal controls [NC], 12 SS and 11 NASH) were studied. LSN quantification procedure was bias correction, boundary detection, segmentation and LSN measurement. LSN scores among three groups and fibrosis grades compared using Kruskal–Wallis H test. Diagnostic accuracy was determined by calculating the area under the receiver operating characteristics (ROC) curve. Mean LSN scores were NC 1.30 ± 0.09, SS 1.54 ± 0.21 and NASH 1.59 ± 0.23 (p = 0.008). Mean LSN scores according to fibrosis grade (F) were F0 1.30 ± 0.09, F1 1.45 ± 0.17 and F2&F3 1.67 ± 0.20 (p = 0.001). The mean LSN score in F2&F3 is significantly higher than that in F1 (p = 0.019). The AUROC curve to distinguish F1 and F2&F3 was 0.788 (95% CI 0.595–0.981, p = 0.019) at a cut-off LSN score greater than 1.48, and its diagnostic accuracy had 0.833 sensitivity and 0.727 specificity. This study developed LSN program and its clinical application demonstrated that the quantitative LSN scores can help to differentially diagnose fibrosis stage in NAFLD.

## Introduction

Nonalcoholic fatty liver disease (NAFLD) is clinically ‘silent liver disease’ such as chronic liver disease and most patients with NAFLD are asymptomatic until development of cirrhosis and hepatic decompensation^1^. NAFLD comprises a wide spectrum of liver damage, ranging from simple macrovesicular steatosis, liver inflammation, fibrosis, and cirrhosis^1–3^. Liver biopsy has been regarded as the reference standard for diagnosing hepatic inflammation, fibrosis and cirrhosis in NAFLD. However, this method has well-known weaknesses with the procedure include the invasive approach, sampling errors, inability to assess the severity of cirrhosis, and complications such as pain, bleeding, infection and rarely death^4,5^. Furthermore, in the NAFLD patients with an initial diagnosis of early stage (compensated) fibrosis or cirrhosis, there are currently no validated non-invasive methods for predicting hepatic decompensation. Therefore, there is an unmet need for widely applicable non-invasive methods to diagnose cirrhosis and advanced liver fibrosis and to predict future risk of hepatic decompensation and death.

Several methods including of clinical and laboratory tests have been used to non-invasively evaluate the hepatic fibrosis and cirrhosis in NAFLD^6–9^. Imaging plays a promising role in assessing fibrosis and cirrhosis, with several methods being employed in clinical practice already, ranging from evaluation of liver morphology to elastography^9–12^. However, conventional imaging modalities including ultrasound (US), computed tomography (CT) and magnetic resonance imaging (MRI) are still limited as imaging biomarkers of NAFLD^13^. Among them, liver MRI has great merits for screening and staging the NAFLD patients with advanced fibrosis and cirrhosis because the imaging modality is widely available (as morphology, texture, elastography, strain imaging, diffusion-weighted imaging, perfusion, hepatocellular function and so on) and no ionizing radiation to patients^10,14^.

The morphological assessment by fibrotic or cirrhotic changes within the liver has been reported in US imaging studies. The levels of diagnostic accuracy varied for prediction of fibrosis stage^15,16^. Recently, liver surface nodularity (LSN) is used to diagnose and stage a variety of liver disease including chronic liver disease, progressive stages of chronic liver disease and cirrhosis, as well as to predict the cirrhosis decompensation and death^17–19^. The LSN can be measured on CT images by using processing program to generate a nodularity score^17–19^. The use of quantitative LSN to stage liver fibrosis has been also applied to CT^18,19^ and MRI^20,21^ with different levels of diagnostic accuracy. Up to date, there is no study focusing on the precision diagnosis according to severity of fibrosis and/or cirrhosis in NAFLD patients using the software-based LSN quantification method.

Therefore, the aim of this study was to develop MRI-suitable LSN quantification program and to refine and validate a new non-invasive imaging biomarker to diagnose advanced liver fibrosis and cirrhosis in the patients with NAFLD.

## Result

### Patient characteristics

**Figure 1:**
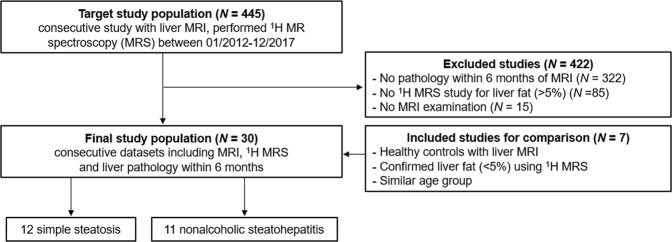
Flowchart of the study population.

Figure 1 shows inclusion criteria of the study population. The averaged enzyme levels and pathological NAS in subjects are shown in Table 1. The serum biochemistry showed significantly different among three groups (Kruskal–Wallis, *p* < 0.05). However, there was no significant difference between both SS and NASH groups as follows: alanine aminotransferase (ALT, *p* = 0.051), aspartate aminotransferase (AST, *p* = 0.060), γ-glutamyl transpeptidase (GGT, *p* = 0.078), fasting glucose (*p* = 0.470), and triglyceride (TG, *p* = 0.231). The steatosis grade, lobular inflammation and NAS scores were significant differences between SS and NASH groups (*p* < 0.01), whereas ballooning scores were not different between the groups (*p* = 0.561).

**Table 1:** Clinical data of serum biochemistry and histopathologic NAS in normal control (NC), SS and NASH groups.

	NC (n = 7)	SS (n = 12)	NASH (n = 11)	*p*-value*
***Demographical characteristics (mean*** ± ***SD)***				
Age (years)	33.3 ± 8.0	41.8 ± 11.2	39.1 ± 13.8	0.289
BMI (body mass index, Kg/m^2^)	21.6 ± 0.8	29.3 ± 2.4	29.2 ± 3.2	<0.001^a^
***Blood chemistry (mean*** ± ***SEM)***^†^				
Aspartate aminotransferase (AST, IU/L)	22.6 ± 1.4	68.6 ± 11.3	103.5 ± 12.3	<0.001^ac^
Alanine aminotransferase (ALT, IU/L)	27.3 ± 2.6	99.2 ± 15.5	185.8 ± 36.0	<0.001^ac^
γ-Glutamyl transpeptidase (GGT, IU/L)	25.6 ± 1.1	68.4 ± 13.5	106.2 ± 17.3	0.001^ac^
Fasting glucose (mg/dL)	80.4 ± 2.5	113.9 ± 9.8	123.0 ± 8.8	<0.001^ac^
Triglyceride (TG, mg/dL)	107.6 ± 6.0	179.1 ± 24.3	243.8 ± 41.4	0.034^a^
***NAFLD activity scores (NAS) (mean*** ± ***SD)***^‡^				
NAS (min. 0 – max. 8)	—	3.6 ± 0.5	6.3 ± 0.8	<0.001
Steatosis grade (min. 0 – max. 3)^d^	—	1.3 ± 0.6	2.5 ± 0.8	0.002
Lobular inflammation (min. 0 – max. 3)^e^	—	1.0 ± 0.0	2.4 ± 0.8	<0.001
Ballooning (min. 0 – max. 2)^f^	—	1.3 ± 0.5	1.5 ± 0.5	0.561
Fibrosis stage (0/1/2/3, n)^g^	—	0/9/3/0	0/2/6/3	—

Abbreviations.– NAFLD, non-alcoholic fatty liver disease; NAS, NAFLD activity score; NASH, nonalcoholic steatohepatitis; SD, standard deviation; SEM, standard error of mean; SS, simple steatosis.

*The difference among three groups was analyzed by the Kruskal–Wallis H test with Mann-Whitney’s *post hoc* test. The significant difference between two groups indicated as follows: ^a^NC vs. SS; ^b^SS vs. NASH; and ^c^NASH vs. NC.

^†^The serum enzyme levels in a normal control group refer to normal range.

^‡^Each NAS score value derived from histology of liver biopsies.

^d^Steatosis grade indicated low- to medium-power evaluation of parenchymal involvement by steatosis as follows: <5%, 0; 5–33%, 1; >33–66%, 2; and >66%, 3.

^e^Lobular inflammation indicated ov^e^rall assessment of all inflammatory foci as follows: no foci, 0; <2 foci per 200x field, 1; 2–4 foci per 200x field, 2; and >4 foci per 200x field, 3.

^f^Ballooning indicated overall assessment of all balloon foci as follows: none, 0; few balloon cells, 1; and many cells/prominent ballooning, 2.

^g^Fibrosis staging indicated overall assessment of fibrotic foci as follows: none, 0; perisinusoidal or periportal, 1; Mild, zone 3, perisinusoidal “delicate” fibrosis, 1a; moderate, zone 3, perisinusoidal “dense” fibrosis, 1b; portal/periportal This category is included to accommodate cases with portal and/or peri portal fibrosis without accompanying pericellular/perisinusoidal fibrosis, 1c; perisinusoidal and portal/periportal, 2; and bridging fibrosis, 3.

### LSN measurements in NAFLD

**Figure 2:**
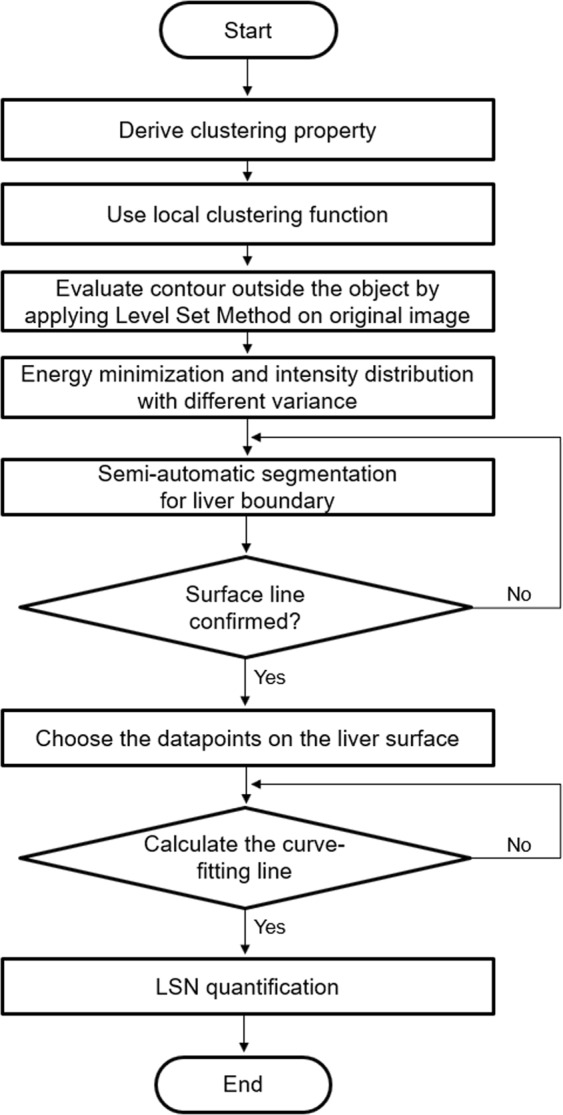
Overall flowchart representing the process for analysis of liver surface nodularity (LSN).

**Figure 3:**
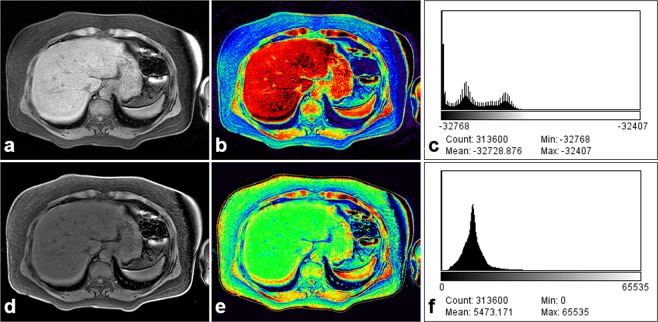
An example showing the bias correction of signal inhomogeneity based on uniformity field in a patient with NAFLD using the NIH color look-up table: (**a**–**c**) without the bias correction and (**d**–**f**) with the bias correction. Black-colored pixels (**b**) on the liver parenchyma were corrected and the shapes of the histogram were smoothed.

**Figure 4:**
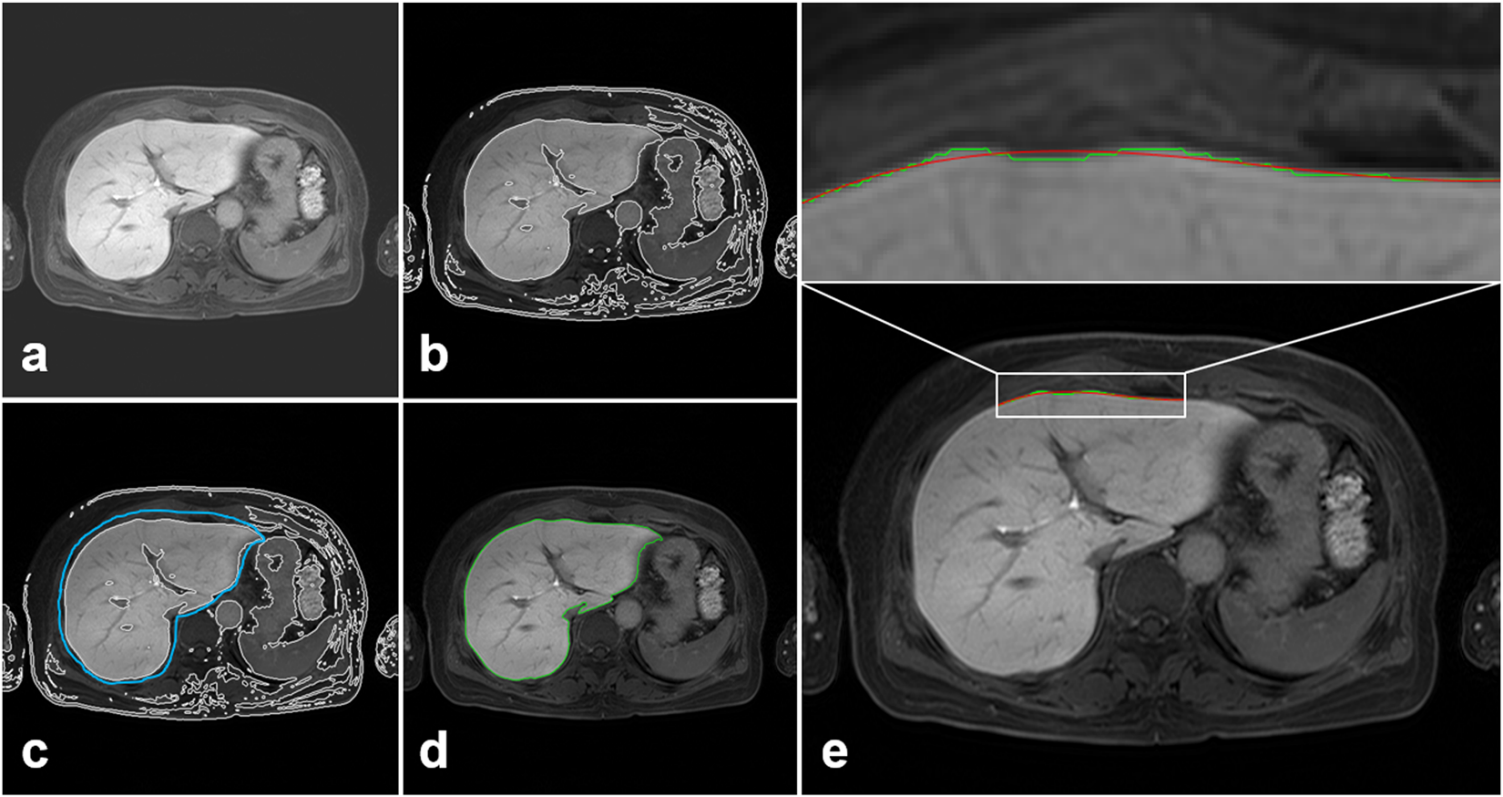
Raw abdominal MR image (**a**), boundary detection after bias correction (**b**), ROI drawing for liver segmentation (**c**, blue-line), final liver surface line (**d**, red-line), and curve-fitting lines (**e**) for LSN quantification on customized LSN software. Note that curve-fitted line (**e**, red-line) used a 4^th^-order polynomial curve shape along the liver surface line (green-line).

MR image data in twenty-three NAFLD patients and seven normal controls were analyzed with developed LSN software. Figure 2 shows the overall processes for LSN analysis. Figure 3 is an example showing the bias correction of signal inhomogeneity. Figure 4 shows the representative abdominal MR image (Fig. 4a), liver boundary detection (Fig. 4b), ROI drawing for liver segmentation (Fig. 4c), final liver surface line (Fig. 4d), and curve-fitting lines (Fig. 4e) for LSN quantification. The median time for LSN measurements was less than 1.5 minutes (range, 0.5–1.3 minutes) and total post-processing time (including bias correction, boundary detection, ROI drawing and curve-fitting) was 3 minutes (range, 2.5–5.3 minutes). LSN scores in three groups were shown in Table 2. The LSN scores were different from three groups (*p* = 0.008). Both SS and NASH patient groups showed higher surface nodularity scores compared with a NC group (*p* < 0.01). However, there was not significantly different between SS and NASH patient groups (*p* = 0.758).

**Table 2:** Liver surface nodularity (LSN) scores in three different groups as normal control (NC), simple steatosis (SS) and NASH.

	NC (n = 7)	SS (n = 12)	NASH (n = 11)	*p*-value^†^
LSN score* [%]	1.30 ± 0.09^7^	1.54 ± 0.21^13^	1.59 ± 0.23^14^	0.008^ac^

Data are presented as mean ± SD [%].

The percentage [%] indicated the coefficient of variation (CV) (CV = (SD/mean of LSN score) × 100).

Note that LSN score in normal controls referred to a reference value.

*LSN in each subject was calculated as arithmetic mean of measurements.

^†^The difference between three groups was assessed by the Kruskal–Wallis H test with Mann-Whitney U test as follows: ^a^NC vs. SS; ^b^SS vs. NASH; and ^c^NASH vs. NC.

**Figure 5:**
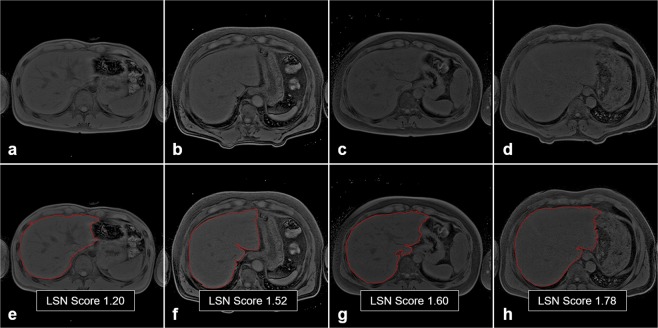
Representative LSN quantification images according to fibrosis grades (F0–F3): F0 (**a**,**e**), F1 (**b**,**f**), F2 (**c**,**g**), and F3 (**d**,**h**). Higher LSN scores are shown with increased surface nodularity in the fibrotic liver.

**Figure 6:**
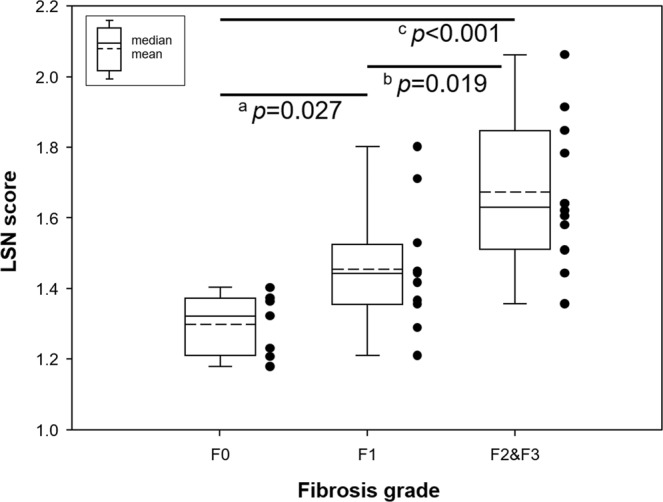
Box plot demonstrating the LSN scores according to different fibrosis degree based on NAFLD activity scores (NAS). The difference between three groups was assessed by the Kruskal–Wallis H test (*p* = 0.001) with Mann-Whitney’s *U* test as follows: ^a^F0 vs. F1 (*p* = 0.027); ^b^F1 vs. F2&F3 (*p* = 0.019); and ^c^F2&F3 vs. F0 (*p* < 0.001). Note that the black-circle dots in the box indicate individual LSN score in each fibrosis grade.

Figure 5 shows the representative LSN quantification images according to fibrosis grades (F0–F3). The LSN scores according to fibrosis grades (F0–F3) were listed in Table 3. Mean LSN scores were 1.30 ± 0.09 for F0 (n = 7), 1.45 ± 0.17 for F1 (n = 11), 1.67 ± 0.20 for F2&F3 (n = 12). Averaged LSN scores are significant different among fibrosis grades (*p* = 0.001). LSN scores ranged from 1.18 to 1.40 (mean 1.30) in the F0, from 1.21 to 1.80 (mean 1.45) in the F1 and from 1.36 to 2.06 (mean 1.67) in the F2&F3, respectively. Figure 6 shows multiple comparisons between fibrosis grades (F0–F3). The significant levels were as follows: F0 vs. F1 (*p* = 0.027), F1 vs. F2&F3 (*p* = 0.019) and F2&F3 vs. F0 (*p* < 0.001), respectively. Therefore, there was significant difference between fibrosis grades in NAFLD based on quantitative LSN scores.

**Table 3:** Comparison of LSN scores according to fibrosis grades.

	F0	F1	F2&F3	*p*-value*
LSN score [%]	1.30 ± 0.09^7^	1.45 ± 0.17^12^	1.67 ± 0.20^12^	0.001^abc^

Data are presented as mean ± SD [%].

The percentage [%] indicated the coefficient of variation (CV) (CV = (SD/mean of LSN score) × 100).

Note that LSN score in normal controls referred to a reference value of fibrosis grade 0 (F0).

The number of patients in F2 and F3 grades merged for statistical analysis.

LSN was calculated as arithmetic mean of measurements.

*The difference between three groups was assessed by the Kruskal–Wallis H test with Mann-Whitney U test as follows: ^a^F0 vs. F1; ^b^F1 vs. F2&F3; and ^c^F2&F3 vs. F0.

### ROC analysis for differential diagnosis according to fibrosis grades

**Figure 7:**
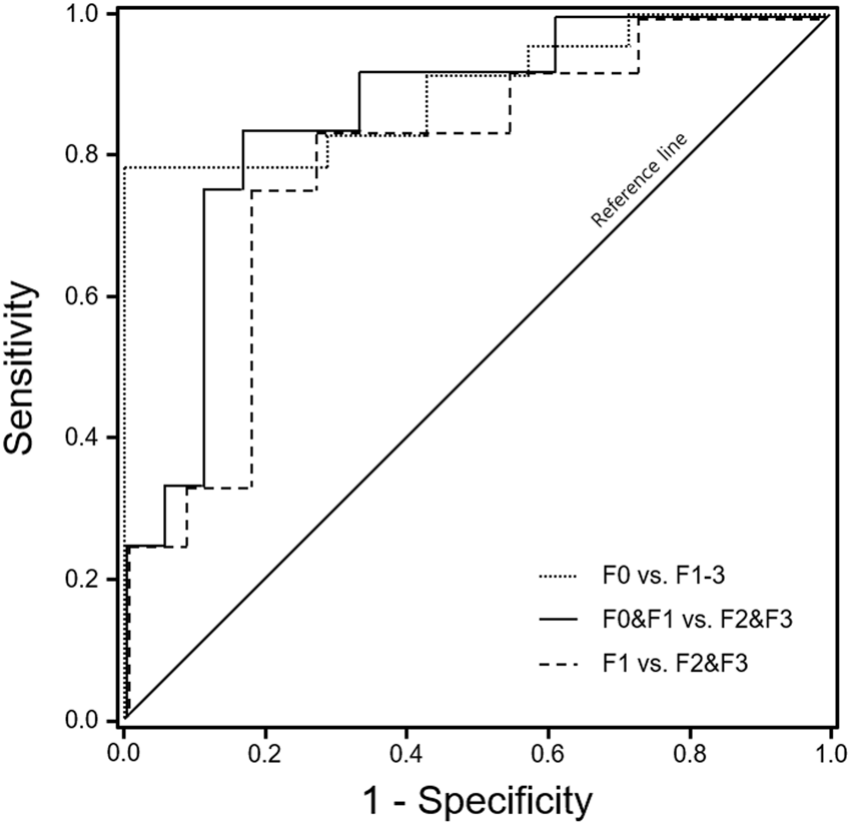
ROC curves of LSN score for the differentiation of fibrosis grades (F). The AUROC curves were F0 vs. F1-3 0.894 (95% CI 0.770–1.000; *p* = 0.002), F0&F1 vs. F2&F3 0.856 (95% CI 0.717–0.996; *p* = 0.001) and F1 vs. F2&F3 0.788 (95% CI 0.595–0.981; *p* = 0.019). The diagnostic accuracy of F0 vs. F1-3 had 0.913 sensitivity and 0.571 specificity at a cut-off LSN score 1.34, F0&F1 vs. F2-F3 was 0.917 sensitivity and 0.667 specificity at a cut-off LSN score 1.43; and F1 vs. F2-F3 was 0.833 sensitivity and 0.727 specificity at a cut-off LSN score 1.48, respectively.

Figure 7 shows ROC curves of LSN score for the differentiation of fibrosis grades and it is summarized in Table 4. The AUROC curves were 0.894 for F0 vs. F1-3 (95% CI 0.770–1.000; *p* = 0.002), 0.856 for F0&F1 vs. F2&F3 (95% CI 0.717–0.996; *p* = 0.001) and 0.788 for F1 vs. F2&F3 (95% CI 0.595–0.981; *p* = 0.019). The diagnostic accuracy of F0 vs. F1-3 had 0.913 sensitivity and 0.571 specificity at a cut-off LSN score 1.34; F0&F1 vs. F2-F3 was 0.917 sensitivity and 0.667 specificity at a cut-off LSN score 1.43; and F1 vs. F2-F3 was 0.833 sensitivity and 0.727 specificity at a cut-off LSN score 1.48, respectively.

**Table 4:** Receiver-operator curve (ROC) analysis for diagnosing fibrosis stage using liver surface nodularity program.

Comparison	Threshold (LSN)	Sensitivity (%)	Specificity (%)	PPV (%)	NPV (%)	AUROC	*p*-value
F0 vs F1-F3	1.34	91.3 (21/23)	57.1 (4/7)	87.5 (21/24)	66.7 (4/6)	0.894	0.002
F0-1 vs F2&F3	1.43	91.7 (11/12)	66.7 (12/18)	64.7 (11/17)	92.3 (12/13)	0.856	0.001
F1 vs F2&F3	1.48	83.3 (10/12)	72.7 (8/11)	76.9 (10/13)	80.0 (8/10)	0.788	0.019

Note.—Data in parentheses are raw data used to calculate percentages. AUROC: area under the receiver-operator curve; F: fibrosis grade; NPV: negative predictive value; PPV: positive predictive value.

### Interobserver agreement

The interobserver variability of LSN scores from 2 observers is summarized in Table 5. There was no significant difference between the averaged LSN values of the 2 observers. Intraclass correlation coefficients (ICCs) were higher than 0.6, indicating good reliability. The ICCs (range: 0.671–0.767) were 0.767 for LSN measurements in NC group, 0.713 for SS and 0.671 for NASH, respectively. Thus, the overall LSN measurements of both observers showed good agreement (*p* < 0.05).

**Table 5:** Interobserver variability in LSN measurements.

	Observer A	Observer B	*p*-value*	Intra-rater reliability (ICC)^†^	95%CI		*p*-value^†^
					Lower bound	Upper bound	
NC	1.30 ± 0.09	1.29 ± 0.11	0.491	0.767	−0.353	0.960	0.049
SS	1.52 ± 0.25	1.55 ± 0.21	0.530	0.713	0.003	0.917	0.025
NASH	1.57 ± 0.19	1.62 ± 0.32	0.477	0.671	−0.222	0.912	0.047
Overall	1.49 ± 0.22	1.52 ± 0.27	0.354	0.778	0.533	0.894	<0.001

Abbreviations: ICC: intraclass correlation coefficient; CI: confident interval.

LSN scores of each observer are presented as means ± SD.

*The differences between both observers in LSN scores were assessed by the Wicoxon signed rank test.

^†^The intra-rater reliability between both observers was assessed by the intraclass correlation (ICC) test.

## Discussion

This study developed semi-automated software for evaluating liver surface nodularity in liver disease and compared the subgroups and fibrosis stages in NAFLD by measuring LSN from retrospective MRI datasets. In our study, abdominal MR images with 3-dimensional T1 high-resolution isotropic volume excitation (THRIVE) pulse sequence demonstrated acceptable accuracy in diagnosing fibrosis grades of NAFLD patients, although the study sample size was small. AUROC-based differentiation between F1 vs. F2&F3 was significant. Smith *et al*.^17^ and Pickhardt *et al*.^18^ studies reported that the diagnostic accuracy of LSN score using MRI and CT images is excellent for predicting fibrosis or cirrhosis (0.910 and 0.959 AUROC). Relatively low AUROC values obtained in our study may be explained by small size of profiled population. It is important to note, however, that current study included only NAFLD patients with ^1^H MR-quantified fibrosis and steatosis. In comparison of patients with minimal (F0-F1) versus readily detectable fibrosis (F2-F3), a relatively high AUROC of 0.857 was reported, thus, confirming previous findings^18^, obtained in patients with chronic end-stage liver disease and/or precirrhotic hepatic fibrosis.

This study investigated the potential variation in LSN measurements and inter-observer assessment. In order to successfully detect signals of liver parenchyma and surface, all MRI data were performed bias correction of field homogeneity before the liver boundary detection. Quantitative LSN scores showed the reliable measurements as an averaged CV value < 15%. The LSN scores measured from two observers were good inter-observer agreement (>0.6), indicating reproducible. Thus, the software-based LSN measurement can be reproducible in clinical MR images.

In NAFLD patients, several studies reported that NASH is histopathologically more severe than simple hepatic steatosis^22,23^. Our pathological and blood chemistry findings were similar to the characterizations as histologically advanced features, a higher NAS value, a higher fibrosis stage, and higher ALT level as contrasted with a NC group (Table 1). The LSN findings identified the LSN differences (higher LSN score) in SS/NASH compared to a NC group. However, there was no significantly different between NASH and SS subgroups. Therefore, the LSN quantification might be difficult to differentiate NASH (induced by hepatocellular inflammation) from simple steatosis (fat accumulation in hepatocyte).

The interesting features in this study are that the LSN scores are significant different among fibrosis grades. Mean LSN score in severe fibrosis grade F2&F3 was significantly higher than those in F0 and F1 (as shown in Table 3). Thus, the LSN quantification can be a non-invasive technique capable of detecting fibrotic changes within the liver parenchyma of NAFLD, and moreover, our data focusing on fibrosis grades of NAFLD can provide the evidence that liver surface nodularity is a useful quantitative imaging biomarker that can be used to diagnose and stage hepatic fibrosis and to predict future hepatic decompensation and death. Major strengths of the technology include the ability to assess previously acquired liver MR or CT images (making it possible to conduct large-scale retrospective population studies), wide availability and frequent use of MR and CT imaging in diagnosing from fibrosis to cirrhosis, rapid processing time (<3 minutes), no requirement for intravenous contrast media and no need for new hardware or special image acquisition procedures. In addition, the non-invasive LSN quantification method on the basis of clinical MR images has the potential in reducing the demand for invasive liver biopsy sampling or additional imaging studies in NAFLD because it can be quantified from acquired image data retrospectively and/or be applied to monitor the disease progression in follow-up data. Moreover, LSN quantification program may be capable of helping the prediction of cirrhosis compensation and death^19^. In previous studies^17,18^, the LSN program has been assessed in the CT images without and with contrast enhancement, it has not yet been applied to MR images. In this point of view, MR imaging may prove potentially useful for quantifying and staging fibrotic/cirrhotic liver. Therefore, our MRI-suitable LSN quantification software will be useful for clinical application, further refinement and validation are essential prior to successful commercialization and clinical implementation of the program for use by radiologist.

Diagnostic accuracy, reproducibility and repeatability in LSN measurements are important criteria to evaluate the performance of a quantitative imaging technique^24^. The LSN scores derived from routine axial 3D-THRIVE images were good reproducibility between two different readers in diagnosing liver fibrosis. Our LSN software can be quantified to MR images in less than 4 minutes. The applicability on prospective and retrospective clinical studies gives LSN program the great merits to predict hepatic fibrosis and to compare disease progression during long-term follow-up studies, especially given high associated reproducibility.

This study included several limitations. First, the study is retrospective, and the study sample size is small due to the inclusion criteria for study population were restricted only NAFLD patients with histopathologically-proven fibrosis staging together with hepatic fat confirmation using ^1^H MR spectroscopy. Second, sub-types of NAFLD were grouped as SS and NASH together with the assessment of hepatic fibrosis stage, it might be presented different degrees or patterns of LSN according to different fibrosis grades. Third, the numbers of subject in each fibrosis grade were low. The shortage of subject number raises issues about whether the patients is small on differences of different fibrosis stage on the basis of LSN scores. Thus, future studies will be necessary to clarify more reproducible and distinctive findings in large NAFLD cohort and population. Another limitation included that other non-invasive markers (imaging or blood based) were not used, thus future study is needed to clarify how this measurement performs compared to what is currently available.

In conclusion, this study developed MRI-suitable LSN quantification program and the LSN measurement is reproducible when applied to MR images in assessing fibrosis stage. The finding demonstrates that the quantitative LSN scores can help to differentially diagnose fibrosis stage in NAFLD.

## Materials and Methods

### Ethics statement

The retrospective research protocol used in this study was approved by the institutional review board (IRB) of Wonkwang University Hospital. Written informed consent was obtained from all study participants for the use of MRI data and electronic health records including pathological information. This study was conducted in accordance with the Helsinki Declaration and Good Clinical Practice.

### Subject population

A total of 30 subjects consisting of 11 patients with histopathologically proven-NASH (mean age 39.1 ± 13.8 yrs), 12 patients with simple steatsosis (SS; mean age 41.8 ± 11.2 yrs) and 7 normal controls (NC; mean age 33.3 ± 8.0 yrs) were recruited for this study from January 2012 to December 2017 (Fig. 1 and Table 1). A normal control group was defined as <5% liver fat content on ^1^H MR spectroscopy (MRS) and NAFLD was defined as >5% liver fat content^25^. The different histological features of NAFLD were assessed by using NAFLD activity scores (NAS). The NAS was calculated by making the sum of the scores for steatosis, lobular inflammation, and ballooning^26^. The NAFLD subgroups are defined SS (NAS < 5) and NASH (NAS ≥ 5) in this study.

The inclusion criteria were as follows: (a) subject age was between 18 and 70 years; (b) alcohol consumption for a 2-year period prior to baseline liver histology: men consuming <20 g per day and women <10 g per day; (c) patients without any active malignant tumors, and chronic/acute liver diseases except obesity or type 2 diabetes; (d) negative patients with viral hepatitis B and C markers; (e) patients without any decompensated liver disease (bilirubin <50 μmol/L, albumin >35 g/L, platelet count >100 × 10^9^/L, international normalized ratio <1.3, no ascites); and (f) patients without any contraindications to MRI examination. All the subjects were excluded secondary etiologies for hepatic fat accumulation^23^.

### Reference standard for diagnosing NAFLD

All liver biopsy specimens in NAFLD were obtained by the percutaneous and/or transjugular approaches (n = 23, 100%). The histologic data were assessed according to the diagnostic criteria of the Pathology Committee as follows^26^: the aggregated NAS [0–8]; the score of each element (steatosis [0–3], lobular inflammation [0–3], ballooning [0–2] and fibrosis scores [0,1,2,3]). NAS system^27^ was used for pathological determination of NAFLD with fibrosis staging (fibrosis grade: F0–F4). Fibrosis grades in subject population were as follows: F0 (total n = 7 (23.3%); NC = 7), F1 (n = 11 (36.7%); SS = 9 vs. NASH = 2), F2 (n = 9 (30.0%); SS = 3 vs. NASH = 6), F3 (n = 3 (10.0%); SS = 0 vs. NASH = 3), and F4 (n = 0 (0.0%)).

### MR imaging acquisition

All MR scans were performed with a 3 T (Tesla) MRI system (Achieva; Philips Medical Systems, Best, The Netherlands) and a 32-channel array coil.

The T1-weighted images (T1WI) were acquired with three-dimensional T1 high-resolution isotropic volume excitation (THRIVE) pulse sequence: TR/TE = 4.2/1.97 msec; field of view (FOV) = 38 × 38 × 14 cm^3^, matrix size = 512 × 512, slice thickness = 0.74 × 0.74 × 2.0 mm^3^, number of slices = 70, number of excitation (NEX) = 2 and scan time = 14 sec. The upper abdominal images were obtained in axial plane and the imaging sequence was triggered to expiration within a single breath-hold. Additional T1- and T2-weighted imaging sequences were acquired in axial, coronal and sagittal sectional planes^28^. Total scan time per subject ranged between 35 and 45 minutes.

### Pre-processing and quantification of MR data for LSN assessment

The LSN quantification software was coded by Matlab program (MathWorks, Natick, Massachusetts). Customized semi-automated post-processing program operates on Windows platform (client version: XP or higher; Microsoft, Redmond, WA). Our LSN program was used the MR images of DICOM format to generate a nodularity score using previously described procedure^17–19^. Figure 2 shows the overall flowchart to develop the algorithm for qualitative and quantitative analysis of LSN. The main algorithm for evaluating the LSN was as follows: the bias correction of field uniformity, the liver boundary detection for drawing the liver reference line, the liver segmentation, and LSN measurement using smooth curve-fitting analysis.

### Bias correction for field uniformity

The settings for optimal window level were automatically or manually adjusted by using the mean signal intensity within liver parenchyma. In order to deal with intensity inhomogeneities on abdominal MR images, we introduced a simple multiplicative-additive model of intensity inhomogeneity^29^. From the physics of images obtained from a variety of imaging modalities (MRI and CT), an obtained image (*I*) can be explained as following Eq. (1)

1 $$I=bJ+n$$

Where *J* is the true image, *b* is the component that reflects the signal intensity inhomogeneity, and *n* is additive noise. The value of component *b* is referred to a value accounting for the field bias. The true image *J* reflects the intrinsic physical properties on the objects being imaged, thus it is assumed to be piecewise constant. The field bias *b* is assumed to be slowly varying. The additive noise *n* can be assumed to be a zero-mean Gaussian noise. This study considers the obtained image *I* as a function defined on continuous domain, followed by the image processing assumption in a previous study^29^. This image model can be described the composition of real-world images, in which signal intensity inhomogeneity (*b*) is attributed to a component of an image^29^.

### Liver boundary detection and liver segmentation for reference line

For the liver boundary detection, this study was used a novel region-based method for liver segmentation as level set method^30^, which was provided the local clustering criterion function with correction with intensity inhomogeneities (Fig. 3). The boundary line on the selected slice liver was produced after the bias correction (Fig. 4b). The liver surface line for LSN quantification was extracted as a reference line and two radiologists (with greater than 20 years’ experience) finally confirmed the liver surface line (Fig. 4d). The boundary detection and segmentation techniques take maximizing the local intensity clustering property and minimizing energy formulation to determine and exclude any existing signal outliers caused by generated systematic artifacts^30^.

### LSN measurement from segmented liver image

Following pre-processing of MR image data, liver parenchyma within confirmed liver surface line were used for the deterministic curve-fitting analysis. Regions of interest (ROIs) for LSN measurement were selected along the boundary of the liver (Fig. 4d). The user input a ROI range across the datapoints of the liver surface line. After input a ROI range, a smooth curve-fitting line (polynomial line shape) was generated on selected ROI dataset (Fig. 4e). Finally, the difference between the liver surface line and a new curve-fitting line was evaluated on a pixel-by-pixel basis. The difference value was squared and then calculated mean value, variation and standard deviation (SD). The final LSN score in individual subject was arithmetically calculated as the mean LSN from the measurements on ROIs. The program exported the final LSN score and the header information of DICOM file to a database.

### LSN analysis in clinical NAFLD patients

The liver MR images in each patient were assessed blindly by two radiologists (HWL, reader A; KHY, reader B) using developed semi-automated LSN software. The radiologists in the liver diagnosis were the experienced abdominal radiologists (>20 years). They had no knowledge of the clinical outcome and access to the readings of the other. To assess the inter-observer variability in the LSN measurements, both radiologists independently assessed the liver images. The overall score of LSN for each patient was calculated as mean score.

Technical details in the LSN measurements are described in a recent paper^17^. In short, the readers pointed out a ROI along the liver surface line on selected slice image. The differences between the detected liver boundary and curve-fitted polynomial line (one of 2^nd^, 3^rd^ and 4^th^-order line shape) on the ROIs was measured on a pixel-by-pixel basis and then averaged for each slice by the program. The overall LSN score was arithmetically calculated as an averaged value of the 4-times measurements. The LSN measurement was well accordant when applied to MRI and CT images. Here, we only used MR image data for LSN quantification for this study. Figure 5 shows the representative images in LSN measurements on the axial MR image.

The MR images were re-analyzed from the same reader after the individual measurements were generated to assure that no sharp turns were falsely provided by the LSN quantification program. At least three and/or four ROI measurements were performed for each subject. A final LSN score was calculated by the program as an averaged value of the individual measurements, with a higher LSN score indicating a higher degree of surface nodularity. The time of processing for quantifying the final LSN score was recorded by the program.

### Statistical analysis

The LSN scores in three independent groups and fibrosis grades were compared using the SPSS version 20.0 program (SPSS Inc., Chicago, IL, USA). The variation in LSN scores was analyzed by the Kruskal–Wallis H test for three groups and the Mann–Whitney *U*-test for intergroup comparisons. Coefficient of variance (CV) was calculated for the variability of LSN measurements^23^. The mean CV values in each group were 7% for NC, 13% for SS, and 14% for NASH (range: 7–14%; average 11.3%). Intra-rater agreement was performed based on the intraclass correlation coefficient (ICC) between the LSN scores. The ICCs were denoted on the basis of the levels of reliability as follows^31^: as poor (<0.4), moderate (0.4 to <0.6), good (0.6 to <0.8), and excellent (0.8 to 1.0).

The diagnostic performance of LSN score according to fibrosis grades was evaluated with receiver operating characteristics (ROC) curve analysis including of the area under the ROC curve (AUROC), sensitivity, and specificity. Statistical significance in all tests was set at two-sided *p*-values less than 0.05.
